# MLH1/PMS2 Expression Could Tell Classical NTRK Fusion in Fluorescence *In Situ* Hybridization Positive Colorectal Carcinomas

**DOI:** 10.3389/fonc.2021.669197

**Published:** 2021-04-29

**Authors:** Yao Fu, Zheng Li, Fuping Gao, Jun Yang, Hongyan Wu, Biao Zhang, Xiaohong Pu, Xiangshan Fan

**Affiliations:** ^1^ Department of Pathology, Nanjing Drum Tower Hospital, The Affiliated Hospital of Nanjing University Medical School, Nanjing, China; ^2^ Ningbo Diagnostic Pathology Center, Ningbo, China; ^3^ Department of Pathology, Gaochun People’s Hospital, Nanjing, China

**Keywords:** colorectal carcinomas, NTRK fusion, MLH1/PMS2, immunohistochemistry, fluorescence *in situ* hybridization, next-generation sequencing

## Abstract

To gain insight into the clinicopathologic profile of colorectal carcinomas harboring oncogenic NTRK fusions based on eastern populations as well as make the best testing algorithm for the screen, we use pan-Trk immunohistochemistry (IHC), fluorescence *in situ* hybridization (FISH) respectively to screen NTRK fusions in a large, unselected cohort of 819 colon cancers; either IHC or FISH positive cases were further detected by next-generation sequencing (NGS). IHC staining was observed in ten (1.22%) cases. FISH positive was observed in 13 (1.59%) cases, and finally, a total of 18 cases were under both a DNA-based and an RNA-based NGS assay. RNA-based NGS was positive in 13 of 18 cases, whereas DNA-based NGS was only positive in three of 18 cases. In total 13 RNA-based NGS NTRK fusion-positive cases, only six cases were pan-TRK IHC positive *versus* 12 were FISH positive. More important, in 13 RNA-based NGS cases only five cases contain the full length of NTRK tyrosine kinase (TK) domain and form the classical fusion chimeras, other six cases only maintain parts of the TK domain and form the sub-classical fusion chimeras, two cases totally miss the TK domain and form the non-classical fusions. For clinicopathologic characteristics, besides the MMR (mismatch repair) status (p = 0.001), there is no difference between the NTRK fusion-positive and negative cases. Nevertheless, classical fusion cases prefer low differentiation (p = 0.001) and different patterns of growth (p < 0.001). Besides, we found all five classical NTRK fusion cases, and only one sub-classical case was harboring MLH1/PMS2 deficiency. When combining FISH and MMR (Mismatch Repair) status, besides one sub-classical case, all five classical fusions were detected, which means MLH1/PMS2 expression could further narrow the classical fusions in FISH NTRK fusion positive cases. Given the low sensitivity and specificity of the pan-Trk antibody, it would be useless to use IHC to screen NTRK fusion-positive CRCs. Combining FISH and MLH1/PMS2 IHC would be a good testing algorithm for the screen effective NTRK fusions. Finally, if patients are going to undergo TRK-based targeted therapy, only RNA-based NGS for detection of the specific fusion could tell the precise rearrangement information.

## Background

Colorectal cancer (CRC) is the third most common cancer worldwide, with more than one million people diagnosed with colorectal cancer every year, and the disease-specific mortality rate is nearly 33% in the developed world and even lower in non-developed countries (1). A range of genomic and epigenomic alterations, most of which are mutations in oncogenes or tumor suppressor genes, have been regarded as targets for colorectal cancer treatment. Nevertheless, colorectal cancer is a subtype of carcinoma characterized by genetic heterogeneity, so every patient advocates different treatments based on the genetic alterations. Except for conventional chemoradiation regimens, molecular target drugs and monoclonal antibodies, such as cetuximab or panitumumab to block EGFR, have also been widely used in colorectal cancer, thereby preventing activation of signal transduction pathways involving RAS, PI3K–AKT, and SRC (2).

Nowadays, neurotrophic receptor tyrosine kinase (NTRK) gene fusion has been found in colorectal cancer and emerged as new promising targets, especially after larotrectinib was approved by the Food and Drug Administration (FDA) of the United States for the treatment of NTRK fusion-positive cancers in 2018. NTRK genes include NTRK1 (chromosome 1q21–q22), NTRK2 (chromosome 9q22), and NTRK3 (chromosome 15q25), which encode three closely related tropomyosin receptor kinase proteins, TrkA, TrkB, and TrkC respectively. Trk proteins, activated by neurotrophins, are expressed in neuronal tissue and contribute to neuronal development, function, and proliferation (3–5). Nevertheless, NTRK fusions also drive the great majority of certain specific rare neoplasms, for example, infantile fibrosarcoma, cellular, mixed congenital mesoblastic nephroma, and secretory carcinoma of the breast and salivary glands with NTRK3 fusions (6–10). And oncogenic NTRK fusions with many other partners also occur at a very low incidence in a wide range of malignancies. Though the prevalence of NTRK fusion is reported as only 0.16–0.31% in colorectal cancer (6, 11), given the high prevalence of CRC, a large number of CRCs driven by NTRK fusions still could benefit from Larotrectinib.

Typically, the fusion chimeras formed when the 5′ region of a gene partner fuses to the 3′ region of the NTRK gene, and these fusions usually expressed constitutively activated tyrosine kinase (11). Detection of oncogenic NTRK fusions has immediate clinical implications, and immunohistochemistry (IHC) has shown significant sensitivity in detecting NTRK fusion specimens. However, given the specificity of IHC, these IHC-positive specimens still need further verification by fluorescence *in situ* hybridization (FISH) or next generation sequencing (NGS). So the consistency of these three technologies needs to be compared. Until now, only limited clinicopathologic data of NTRK fusion positive CRCs are available (9, 12–18), the clinicopathologic profile of primary tumors harboring oncogenic NTRK fusions remains to be elucidated.

In this study, a large, unselected cohort of 819 colon cancers was screened for NTRK fusion positive cases. Using IHC, FISH, and NGS we want to find the best testing algorithm. During the course of the study, clinicopathologic, immunohistochemical, and molecular genetic features of NTRK fusion positive tumors were studied in detail.

## Patients

We developed a cohort of unselected patients undergoing surgical resection for CRC by searching the computerized database of the Department of Pathology, Nanjing Drum Tower Hospital, Nanjing, China for all cases between 2015 and 2020. The inclusion criteria were as follows: 1) pathologically diagnosed adenocarcinoma, mucinous adenocarcinoma, or high grade neoplasia according to the latest WHO classification; 2) complete clinical and pathological data. Exclusion criteria included: 1) extracolonic and appendiceal location; 2) tumors undergoing biopsy alone or treated endoluminally; 3) preoperative local or systematic anticancer neoadjuvant therapy; 4) incomplete clinical data. Patients’ consent for surgical resection and clinical research was obtained in all cases before the surgical resection. The entire cohort was annotated for clinicopathological details including stage, grade, MMR protein, and KRAS/NRAS/BRAF genetic status.

## Immunohistochemistry

Pan-Trk IHC and DNA mismatch repair (MMR) proteins including MutL Homolog 1 [MLH1], PMS1 Homolog 2 [PMS2], MutS Homolog 2 [MSH2], and MutS Homolog 6 [MSH6] IHC were performed for all of the cases. All next generation sequencing (NGS) proved NTRK translocation positive cases further underwent CD3, CD8, and PD-L1 IHC. Representative 4 μm serial sections of the tumor were prepared from 10% FFPE tissue blocks for IHC. Briefly, all slides were exposed to 3% hydrogen peroxide for 10 min to block endogenous peroxidase activity. Pan-Trk antibody (Clone: EPR17341, Abcam, USA), DNA mismatch repair (MMR) proteins (MutL Homolog 1 [MLH1] (Clone: ES05, Dako Denmark A/S, Denmark), PMS1 Homolog 2 [PMS2] (Clone: EP51, Dako Denmark A/S, Denmark, Dako Denmark A/S, Denmark), MutS Homolog 2 [MSH2] (Clone: FE11, Dako Denmark A/S, Denmark), and MutS Homolog6 [MSH6] (Clone: Pu29,Dako Denmark A/S, Denmark)), PD-L1 (Clone: 22C3, Dako Denmark A/S, Denmark), CD3 (Clone: F7.2.38, Dako Denmark A/S, Denmark), CD8 (Clone: C8/144B, Dako Denmark A/S, Denmark) incubated with tumor sections in a humidified chamber at 4°C overnight, followed by the secondary anti-mouse peroxidase-conjugated secondary antibody (EnVisionTMDetection Kit, Dako, Glostrup, Denmark) or anti-rabbit peroxidase-conjugated secondary antibody (EnVisionTM Detection Kit, Dako, Glostrup, Denmark) at 37°C for 30 min. Both negative (without the primary antibody) and positive controls were carried out in each run. Cytoplasmic staining intensity was considered positive.

The resulting score was calculated by multiplying the staining intensity (0 = no staining, 1 = mild staining, 2 = moderate staining, and 3 = strong staining) by the percentage of immunoreactive tumor cells (0 to 100). The immunostaining result was considered to be 0 or negative when the score was <25; 1+ or weak when the score was 26–100; 2+ or moderate when the score was 101–200; or 3+ or strong when the score was 201–300. The results of IHC were interpreted independently by two pathologists who were blinded to all clinical and pathological data.

## Fluorescence *In Situ* Hybridization Testing

All cases underwent FISH testing. FISH testing for NTRK gene rearrangements used the NTRK1/2/3 Dual Color Break Apart Probe (Anbiping, China) respectively. FISH was performed according to the manufacturer’s instructions. For all NTRK1/2/3, red probes were labeled 5′end, and green probes were labeled 3′end (containing TK domain), whereas, the transcription direction of NTRK3 was opposite from NTRK1 and NTRK2. In our study, any case with signal break-apart or single red/green was regarded positive. The threshold of positive specimens for gene rearrangement was considered 15% break-apart signals, or the same percentage with single green/red signals (19).

## DNA and RNA Extraction

DNA and RNA were recovered from formalin-fixed paraffin-embedded colon carcinoma specimens using a Max-well RSC instrument and DNA or RNA FFPE Kit (Promega, Madison, WI), according to the manufacturer’s protocols.

## KRAS, NRAS and BRAF Genetic Mutation Testing

Genomic DNA was extracted from formalin-fixed paraffin-embedded (FFPE) tissues using AmoyDx FFPE DNA Kit (Amoy Diagnostics Co. Ltd, China) according to the manufacturer’s protocol. KRAS (exons 2, 3 and 4) and NRAS (exons 2, 3 and 4) mutations were detected using AmoyDx KRAS/NRAS Mutation Detection Kit and BRAF V600E were detected using AmoyDx BRAF Mutation Detection Kit (Amoy Diagnostics Co. Ltd, China) using ABI 7500(Applied Biosystems, USA) according to the manufacturer’s instruction, and the results were analyzed in accordance with the manufacturer’s manuals.

## ArcherDx Assay (RNA-Based Next-Generation Sequencing)

Target-specific libraries for next-generation sequencing (NGS) were constructed using Archer Universal RNA Reagent Kit v2 (ArcherDx, Boulder, CO). Library sequencing was accomplished using a MiSeqDx instrument (Illumina, San Diego, CA). NGS data were analyzed using the Archer Analysis Pipeline Virtual Machine (https://archerdx.com).

## Ion Torrent NGS (DNA-Based Next-Generation Sequencing)

DNA-based NGS was performed by Macrogen USA (Rockville, MD) using the Ion Torrent (Life Technologies/Thermo Fisher Scientific, Waltham, MA) NGS platform. Bioinformatics analysis of NGS data was processed by Torrent Server Suite 4.2 and sequences aligned to human genome reference sequence HG-19 (The Genome Reference Consortium). The FATHMM (Functional Analysis Through Hidden Markov Models), SIFT (Sorting Intolerant from Tolerant), and PolyPhen (Polymorphism Phenotyping) scores predicting functional consequences of coding variants were either obtained from the COSMIC (Catalogue of Somatic Mutations in Cancer) at https://cancer.sanger.ac.uk or assessed during bioinformatic analysis.

## Statistical Analysis

All data analyses were performed using SPSS 19.0 software (SPSS Inc., Chicago, Illinois, US). Fisher’s exact test was used for categorical data, and the Student t-test was used for continuous data. Analysis of variance or the Kruskal–Wallis rank sum test was used to compare differences among different groups. The Chi-square or Fisher’s exact test was utilized for comparison of ratios. Differences were considered to be statistically significant when p values were less than 0.05.

## Results

### Consistency of IHC, FISH and NGS

Pan-TRK IHC staining was observed in ten (1.22%) of 819 cases, FISH positive was observed in 13 (1.59%) of 819 cases, and finally a total of 18 cases with either IHC or FISH positive were under both a DNA-based and an RNA-based NGS assay. RNA-based NGS was positive in 13 of 18 cases; DNA-based NGS was only positive in three of 18 cases listed as two TPM3-NTRK1 fusions and one TPR-NTRK1 fusion. Details of immune and molecular characteristics of IHC, FISH, or NGS positive cases were listed in **Table 1**. In ten immunohistochemical staining positive cases, five cases presented nuclear staining, four cases presented cytoplasmic staining, and only one case presented nuclear membrane staining. In 13 FISH positive cases, the signal modes presented three different types: break-apart signals, single red or single green signals. In our study, any type of these three signal patterns was regarded as positive. In the total of ten pan-TRK IHC positive cases, only six cases were also proved positive by RNA-based NGS, and the other four IHC positive cases were proved negative by RNA-based NGS. Whereas 12 of 13 FISH positive cases were proved as NTRK fusion by RNA-based NGS. Comparatively, FISH was much more sensitive than pan-TRK IHC. Nevertheless, in a total of 13 RNA-based NGS cases, only five cases formed the classical fusion chimeras; the other six cases which only maintain parts of the tyrosine kinase (TK) domain of NTRKs were not sure for the therapeutic effect when patients under NTRK fusion-based target treatment. And the remaining two cases totally missed the TK domain of NTRKs and were regarded as non-classical fusions.

**Table 1:** Immune and molecular characteristics of IHC, FISH or NGS NTKR fusion-positive cases.

Case No.	RNA-based NGS result	Fusion type	Classical fusion	DNA-Based NGS result	Fusion type	IHC result	Pan-TRK-IHC pattern			FISH result	FISH-signal type		
							Nuclear	Nuclear memberane	Cytoplasmic		1R1 G1F	1R	1G
**1**	+	TPM3(E8)- NTRK1(E8)	+	+	TPM3(E8)- NTRK1(E8)	+	+			+	+		
**2**	+	TPM3(E8)- NTRK1(E8)	+	+	TPM3(E8)- NTRK1(E8)	+	+			+	+		
**3**	+	TPR (E21)- NTRK1(E8)	+	+	TPR (E21)- NTRK1(E8)	+	+			+	+		
**4**	+	5’-telomere- NTRK1(E8)	+	NT	NT	+			+	+	+		
**5**	+	NTRK1(E12)- ALLC(E1)	+/−	NT	NT	+			+	−			
**6**	+	NTRK1(E7)- ITGB5(E4)	−	NT	NT	–				+	+		
**7**	+	NTRK1(E7)- LPP (E1)	−	NT	NT	+	+			+		+	
**8**	+	APBB1IP(E10)- NTRK2(E15)	+	NT	NT	−				+			+
**9**	+	NTRK2(E15)- FAM110B(E5)	+/−	NT	NT	−				+		+	
**10**	+	ALLC (E12)- NTRK3(E15)	+/−	NT	NT	−				+		+	
**11**	+	CCDC73(E2)- NTRK3(E15)	+/−	NT	NT	−				+			+
**12**	+	NTRK3(E14)- PBX1 (E1)	+/−	NT	NT	−				+		+	
**13**	+	NTRK3(E15)- HOXC13 (E12)	+/−	NT	NT	−				+	+		
**14**	−	NT	NT	NT	NT	−				+			+
**15**	−	NT	NT	NT	NT	+	+			−			
**16**	−	NT	NT	NT	NT	+		+		−			
**17**	−	NT	NT	NT	NT	+			+	−			
**18**	−	NT	NT	NT	NT	+			+	−			

NT, Not detected.

### Fusion Modes of NTRKs in CRCs

**Figure 1:**
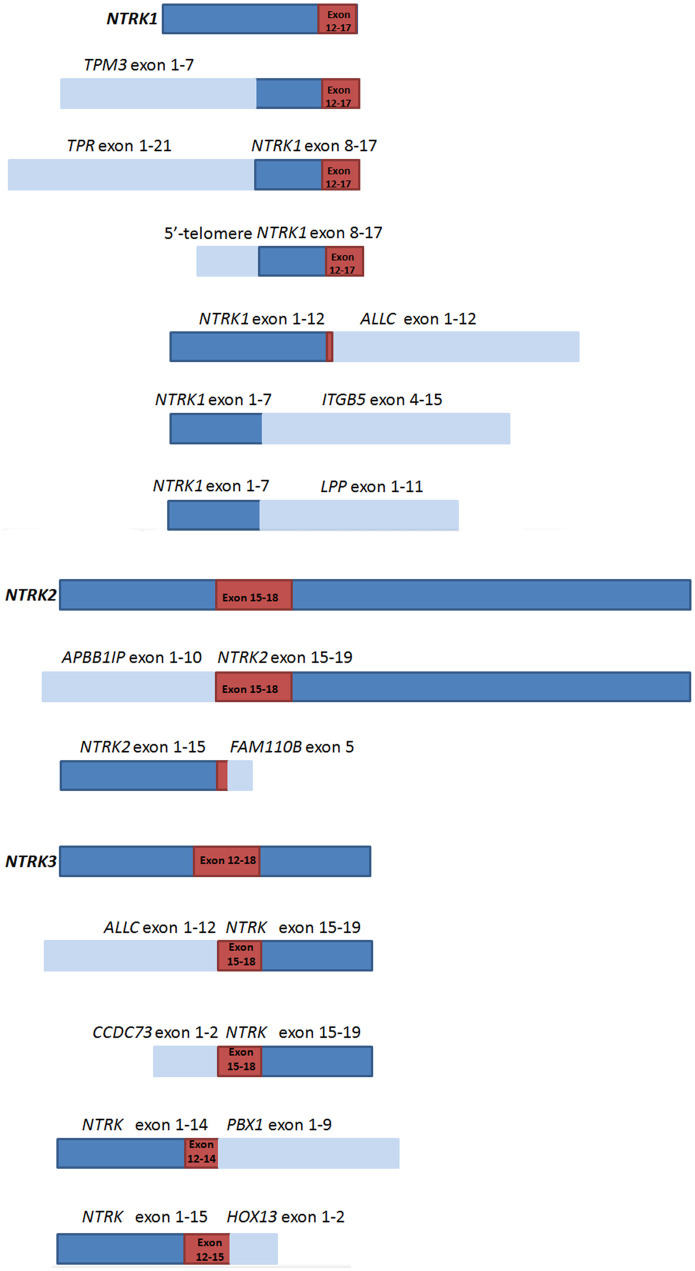
Schematic representation of NTRKs and NTRK fusion chimeras. 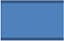 represents NTRK genes, 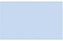 represents fusion partners, 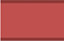 represents domains that encode the tyrosine kinase of NTRKs.

Whole transcriptome high-throughput sequencing of tumor specimens is regarded as the most effective method to screen fusion oncogenes. So we set RNA-based NGS as the “golden standard” and try to find fusion partners as well as analyze the fusion modes of specimens carrying NTRK translocations in CRCs. Based on the breakpoints and fusion modes, NTRK fusion chimeras can be divided into three types: fusions containing the full length of NTRK tyrosine kinase (TK) domain and forming the classical chimeras, fusions only maintaining parts of the TK domain and forming the sub-classical chimeras, and fusions totally missing the TK domain and forming the non-classical fusions. As larotrectinib and other Trk inhibitors are a set of tyrosine kinase inhibitors, only patients carrying the TK domain of NTRK fusions could be regarded as effective fusions that respond to Trk inhibitor treatment. In total 13 RNA-based NGS cases (NTRK1 fusion n = 7, NTRK2 fusion n = 2, NTRK3 fusion n = 4) only five cases [TPM3-NTRK1 (n = 2), TPR-NTRK1, 5′-telomere-NTRK1, ALLC-NTRK3) formed the classical fusion chimeras; the other six cases which only maintain parts of the tyrosine kinase (TK) domain of NTRKs (NTRK1-ALLC, NTRK2-FAM110B, ALLC-NTRK3, CCDC73-NTRK3, NTRK3-PBX1, NTRK3-HOXC13) were regarded as sub-classical fusions, and the remaining two cases (NTRK1-LPP, NTRK1-ITGB5) totally missing the tyrosine kinase (TK) domain of NTRKs were regarded as non-classical fusions. In theory, we speculated that classical fusions were sensitive to NTRK fusion targeted drugs, and non-classical fusions were non-sensitive to NTRK fusion targeted drugs. However, whether sub-classical fusions are sensitive to target treatment is unknown. The diagrammatic sketch of the classical, sub-classical, and non-classical NTRK fusions were shown in **Figure 1**. Except for TPM3 and TPR, the other fusion partners were firstly reported in our studies.

### IHC Staining Pattern of NTRK Positive CRCs

**Figure 2:**
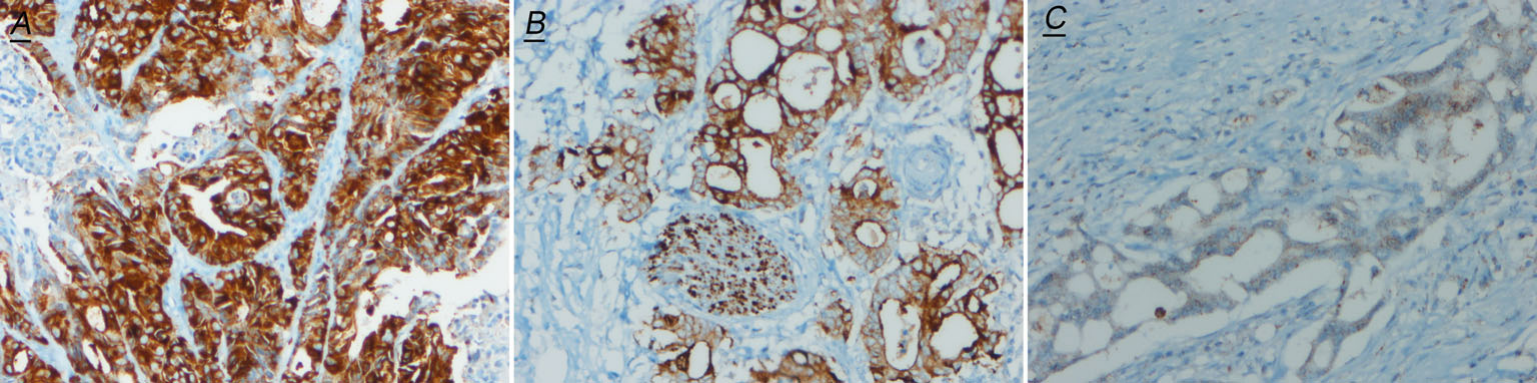
Representative images of pan-Trk IHC (200×) positive cases. **(A)** shows nuclear immunostaining; **(B)** shows nuclear membrane immunostaining; **(C)** shows cytoplasmic immunostaining.

As described previously, there were three immunostaining patterns in IHC positive cases: one showed nuclear positive staining (n = 5); the second showed diffuse cytoplasmic staining in all neoplastic cells (n = 4), and the last pattern was noted in the nuclear membrane (n = 1) (**Figure 2**). In five nuclear positive staining cases, three of them were proved classical NTRK fusions, one was proved a non-classical NTRK fusion, and one was detected NTRK fusion negative by NGS. In four diffuse cytoplasmic staining cases, one was proved classical NTRK fusion, one was proved sub-classical NTRK fusions, and the other two cases were detected NTRK fusion negative by NGS. One nuclear membrane staining case was also detected NTRK fusion negative by NGS (**Table 1**). Although all these three patterns were regarded positive for IHC diagnosis staining, not all of these patterns were proved NTRK fusion by NGS, and nuclear staining with more cases (3/5) proved NTRK fusion by NGS than cytoplasmic staining (1/4) and nuclear membrane staining (0/1).

### FISH Pattern of NTRK Positive CRCs

**Figure 3:**
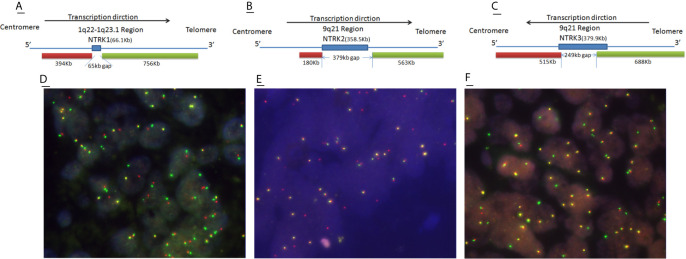
Schematic representation of NTRK break-apart FISH probes and representative images of FISH positive cases. **(A**–**C)** 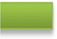 and 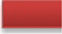 represent green and red fluorescence probes respectively. **(D)** Break-apart (1red1green1fusion) signal, **(E)** red signal, **(F)** green signal.

For all NTRKs, red fluorescence probes were labeled 5′end and green probes were labeled 3′end (containing TK domain). For NTRK1/2, the 5′ region of gene partner fuses to the 3′ region of NTRK gene, whereas for NTRK3, the transcription direction was opposite from the NTRK1 and NTRK2 (**Figures 3A–C**). So in theory, for NTRK1/2 break-apart single and signal green single, for NTRK3 break-apart single and signal red single, are all maintaining the TK domain and should assemble effective fusions. However, compared with NGS, even classical break-apart does not fully mean the classical fusion chimeras. So in our study, any case with a break-apart signal or single red/green signal was regarded as positive (**Figures 3D–F**). And we found that cases with single signals missing the kinase domain of NTRK in theory were often proved as sub-classical fusions by NGS. The signals of FISH representation were as follows: break-apart signal (NTRK1 n = 5; NTRK2 n = 0; NTRK3 n = 1), single red signal (NTRK1 n = 1; NTRK2 n = 1; NTRK3 n = 2), and single green signal (NTRK1 n = 1; NTRK2 n = 0; NTRK3 n = 2). For NTRK1 FISH, one case with break-apart signal and one case with only red signal were both detected by NGS with non-classical fusion [NTRK1(E7)-ITGB5(E4) and NTRK1(E7)-LPP (E1)]. For NTRK2 FISH, there was no classical break-apart signal; one case with only green signal was detected with classical fusion [APBB1IP(E10)-NTRK2(E15)], and the other with only red signal was detected with sub-classical fusion [NTRK2(E15)-FAM110B(E5)]. For NTRK3 FISH, all four positive cases were proved sub-classical fusions by NGS. Two cases with only the red signal by FISH were detected with ALLC (E12)-NTRK3 (E15) and NTRK3 (E14)-PBX1 (E1) fusion respectively. One case with break-apart signal was detected with NTRK3 (E15)-HOXC13 (E12) fusion, and one case with only a green signal was detected with CCDC73 (E2)-NTRK3 (E15). It’s worth noting that one case with FISH 1G signal pattern was proved NTRK fusion negative by NGS (**Table 1**).

### Clinicopathological Characteristic of NTRK Fusion Positive and Negative CRCs

**Figure 4:**
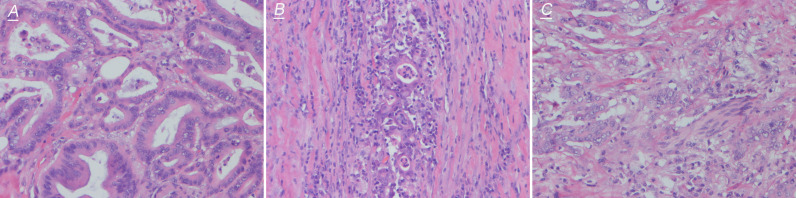
Representative images of hematoxylin–eosin staining (200×) of NTRK effective fusion cases. **(A)** shows high differentiation, **(B)** shows medium differentiation, and **(C)** shows low differentiation.

The comparison of the clinicopathological characteristics of NTRK translocation positive cases, classical fusion cases, sub-classical fusions, non-translocation cases is summarized in **Table 2**. Besides MMR (mismatch repair) status (p = 0.001), there was no significant difference in age, tumor size, tumor differentiation, AJCC stage, TNM stage, histological grade, a pattern of growth, and KRAS/BRAF/NRAS status between translocation-positive and negative cases. However, when referring to classical fusions, same as in the North American study, we found that patients carrying NTRK classical fusions preferred poor differentiation cohort (p = 0.001) (**Figure 4**). In the total of five classical cases, three displayed ulcerous, one displayed pushing, and one displayed an infiltrative pattern of growth. Compared with non-NTRK fusion cases, there was a significant difference in tumor pattern of growth (p < 0.01) between classical and non-fusion cases. For MMR status, all five classical and one sub-classical NTRK fusion cases were harboring mismatch repair defection (p < 0.01).

**Table 2:** Cliniopathological characteristic of NTRK fusion-negative (n = 806) and different NTRK fusion-positive CRCs.

Variables	NTRKs fusion negative(n = 806)	RNA-based NTRKs fusion positive (n = 13)	RNA-based NTRKs classical fusion (n = 5)	RNA-based NTRKs sub-classical fusion (n = 6)	*p1 value*	*p2 value*	*p3 value*
**Gender(Male/Female)**	476/330	7/6	2/3	4/2	0.779	0.406	1.000
**Age(mean)**	61.44(22–89)	63.08(38–78)	62.6(38–78)	63.0(52–75)	1.000	1.000	1.000
**Tumor Location**							
**(left/right)**	585/221	10/3	2/3	6/0	1.000	0.132	0.198
**Tumor maximum** **dimension (cm)**	2.65(0.1–9.0)	2.32(0.8–8.0)	2.68(0.8–6.0)	2.13(0.8–8.0)	0.963	0.875	0.896
**Histological grade**					0.706	0.875	0.852
**Adenocarcinoma**	765	13	5	6			
**Mucinous adenocarcinoma**	36	0	0	0			
**High Grade neoplasia**	5	0	0	0			
**Pattern of growth**					0.052	0.000*	0.178
**Ulcerous**	529	7	3	3			
**Pushing**	250	4	1	2			
**Infiltrative**	7	1	1	0			
**Pushing- ulcerous**	20	1	0	1			
**Differentiation**					0.040	0.001*	0.811
**High/Medium/Low**	15/603/188	1/6/6	1/1/3	0/4/2			
**AJCC stage 8th Ed**					0.280	0.495	0.926
**Ⅰ/Ⅱ/Ⅲ/Ⅳ**	84/285/405/32	3/6/4/0	1/3/1/0	1/2/3/0			
**T Stage**	21/85/642/58	1/2/10/0	1/0/4/0	0/1/5/0	0.482	0.092	0.847
**N Stage (N_O_/N_1_/N_2_)**	373/285/148	9/4/0	5/0/0	3/3/0	0.142	0.056	0.477
**M Stage(M_0_/M_1_)**	775/31	13/0	5/0	6/0	1.000	1.000	1.000
**MMR status**					0.001*	0.000*	0.464
**pMMR/dMMR**	727/79	7/6	0/5	5/1			
**KRAS status**					0.744	1.000	1.000
**WT/Mutation**	628/178	11/2	4/1	5/1			
**NRAS status**					0.077	0.031	1.000
**WT/Mutation**	802/4	12/1	4/1	6/0			
**BRAF status**					1.000	1.000	1.000
**WT/Mutation**	795/11	13/0	5/0	6/0			
**KRAS/BRAF/NRAS status**					1.000	0.599	1.000
**WT/Mutation**	613/193	10/3	3/2	5/1			

p1 represents difference compared NTRK fusion negative with RNA-based NTRK fusion groups.

p2 represents difference compared NTRK fusion negative with RNA-based NTRK classical fusion.

p3 represents difference compared NTRK fusion negative with RNA-based NTRK sub-classical fusion. *Statistically significant.

### Clinical, Pathological, Immunological and KRAS/NRAS/BRAF Genetic Status of NTRK Positive CRCs

Detailed clinical, pathological, immunological, and KRAS/NRAS/BRAF genetic statuses of 13 RNA-based NTRK fusion-positive cases were presented in **Table 3**. NTRK fusion-positive patients almost showed gender balance with six females and seven males. And classical fusions showed three females and two males. As to sub-classical NTRK fusions, males (n = 4) were more than females (n = 2). In 13 RNA-based NTRK fusion-positive cases, three were diagnosed as AJCC stage I, six were stage II, and four were stage III. Seven tumors presented an ulcerous pattern of growth, four were pushing, one infiltrative, and one pushing-ulcerous. Only one tumor showed high differentiation, six tumors showed medium differentiation, and six tumors showed low differentiation. Six of 13 (46.15%) NTRK fusion colon cancers revealed a loss of MLH1/PMS2 expression, indicating MMR protein deficiency; none of the cases was seen to have MSH2 or MSH6 deficiency. Three cases showed PD-L1 expression; one was an NTRK classical fusion case, and two were NTRK non-classical fusion cases. CD3 and CD8 expression was variable and presented in all of NTRK fusion tumors; in most of the cases, expression in the infiltrate margin was higher than in the central tumor areas. Tumor budding can be seen in six tumors, but they were all low grade (1–4 buds/per count). Tertiary lymphoid structures (TLSs) which are considered to be important sites for the initiation and/or maintenance of local and systemic anti-tumor immune response, were present in seven of 13 cancers; six cases show one TLS under 200 magnification, and one shows two TLSs under 200 magnification.

**Table 3:** Clinical, pathological, immunological and molecular characteristics of 13 NTRKs fusion Positive CRCs.

Case No.	classical fusion	Gender	Age	TNM Stage	Pattern of growth	Differentiation	Tumor budding	TLS	Mismatch repair proteins expression	PD-L1	CD3CT	CD3IM	CD8CT	CD8IM	*KRAS/NRAS/BRAF* Status
1	+	Male	55	II	Infiltrative	Low	1	1	MLH1 deficiency	NT	240	1080	100	150	WT
2	+	Female	78	III	Ulcerous	Low	0	1	PMS2 deficiency	NT	65	400	130	400	WT
3	+	Male	69	II	Ulcerous	Low	0	1	PMS2 deficiency	20%	150	500	140	240	WT
4	+	Female	73	I	Pushing	High	0	0	PMS2 deficiency	NT	50	190	35	75	*NRAS* mutation
5	+/−	Female	75	I	Pushing	Medium	0	0	pMMR	NT	100	160	10	45	WT
6	−	Male	63	II	Ulcerous	Low	1	1	pMMR	5%	110	80	5	40	WT
7	−	Female	66	I	Pushing	Medium	0	1	pMMR	20%	140	120	5	45	WT
8	+	Female	38	II	Ulcerous	Medium	0	1	MLH1/PMS2 deficiency	NT	250	150	20	80	*KRAS* mutation
9	+/−	Male	53	III	Pushing	Low	1	0	pMMR	NT	120	420	25	400	WT
10	+/−	Male	52	II	Ulcerous	Medium	0	2	pMMR	NT	70	125	55	70	WT
11	+/−	Female	74	III	Ulcerous	Medium	1	0	pMMR	NT	120	210	100	150	WT
12	+/−	Male	63	III	Pushing- Ulcerous	Medium	1	0	pMMR	NT	20	240	25	50	*KRAS* mutation
13	+/−	Male	61	II	Ulcerous	Low	1	0	MLH1/PMS2 deficiency	NT	90	400	25	75	WT

TLS, Tertiary lymphoid structures; CT, central tumor; IM, infiltrate margin; +, classical fusion; +/−, sub-classical fusion; -, Non- classical fusion; NT, Not detected.

### MLH1/PMS2 Expression and KRAS/NRAS Genetic Status in NTRKs Fusion Positive Cases

All 819 cases underwent MLH1, PMS2, MSH2, and MSH6 IHC and KRAS/NRAS/BRAF genetic mutation testing. We found classical NTRK fusions were highly enriched in MLH1/PMS2 deficient colorectal carcinomas. In a total of five classical NTRK fusion cases, all were carrying MLH1/PMS2 deficiency, whereas only one of six sub-classical NTRK fusion cases were carrying MLH1/PMS2 deficiency. As to KRAS/NRAS genetic mutation, there was no significant difference between RNA-based NGS NTRK positive and negative cases. However, when narrowed to classical NTRK fusions, two of five classical NTRK fusion cases were with KRAS (n = 1) or NRAS (n = 1) mutation *versus* one case with KRAS mutation in sub-classical NTRK fusions. However, there was no significant difference in KRAS/NRAS/BRAF status between classical NTRK fusions and NTRK fusion negative cases.

### Testing Algorithm for NTRK Fusions in CRCs

**Figure 5:**
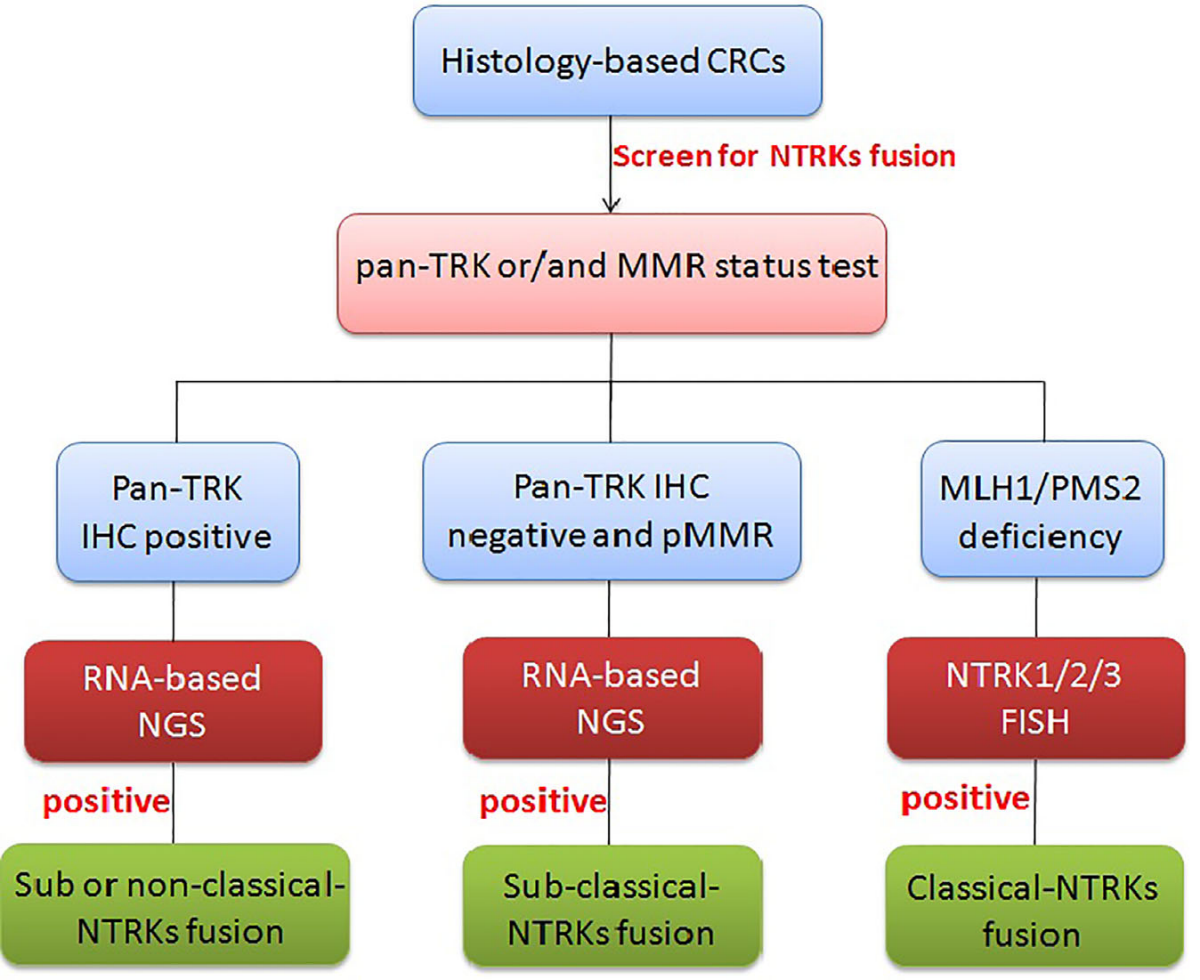
Diagnostic algorithm for NTRK testing in CRCs. In CRCs, pan-TRK IHC as the primary screen tool and MMR IHC can be used as an initial screen, but if pan-TRK IHC positive or/and MLH1/PMS2 deficiency, further FISH/NGS should be undergone to confirm. MLH1/PMS2 deficiency with FISH positive usually means classical NTRK fusions. If neither of pan-TRK IHC positive nor MMR deficiency, RNA-level fusion testing should further be used to select the sub-classical NTRK fusions.

While the importance of identifying patients that could benefit from NTRK fusion-based targeted therapy cannot be understated, accuracy and economic considerations should also be taken into account when creating testing algorithms and guidelines. Although the sensitivity and specificity are very low compared with NGS, pan-TRK IHC is pretty much cheaper and more feasible than other methods; especially pan-TRK IHC positive cases have a good chance to be classical fusions, so we recommend pan-TRK IHC as the primary screen tool. As NTRK fusions (especially classical fusions) were mostly narrowed to MLH1/PMS2 deficiency cases and DNA mismatch repair (MMR), IHC testing was regularly undergone by all CRC patients. MMR status could also be the first step to screen potential NTRK fusion-positive patients. If pan-TRK IHC positive or/and MLH1/PMS2 deficiency, we recommend using FISH/NGS to further confirm. Because FISH lack the ability to identify classical, sub-classical, and non-classical fusions, other information must support to mark the classical fusions. FISH testing combined with DNA mismatch repair (MMR) IHC testing almost totally matches the classical fusions cases; it would be the best combination for selecting classical NTRK fusions. However, with sub-classical NTRK fusions with negative pan-TRK IHC and FISH or with pMMR, NGS is the only method to select these types of patients for molecular target treatment. Overall, a comprehensive test algorithm is using pan-TRK IHC and DNA mismatch repair (MMR) IHC testing for preliminary screening and combining FISH for enriching classical fusions. Anyway, if NGS is available, it must a finally and definitively clinically validated methodology to tell the precise rearrangement information (**Figure 5**).

## Discussion

Nowadays, NTRK fusion has emerged as a popular target for treatment, especially after larotrectinib was approved by the FDA for the treatment of NTRK fusion-positive cancers in 2018. However, except for ETV6–NTRK3 fusion in congenital mesoblastic nephroma (cellular or mixed subtypes) and infantile fibrosarcomas with a prevalence of >90% in selected series of patients (20–23), the prevalence of NTRK fusion is found at much lower frequencies (5–25 or <5%) in other more common tumors such as breast, lung, and colorectal cancers. In colorectal cancers (CRCs), the prevalence of NTRK fusion has been reported only 0.16–0.3% based on western countries (6, 11). Our studies based on the Chinese population showed that NTRK fusion occurred in 13 of 819 patients with a 1.59% incidence rate which was more than the prevalence in western countries. However, when we further analyzed the break-apart point and fusion modes of these 13 cases, we found that only five of them formed the classical chimeras retaining the full tyrosine kinase domain of NTRKs. So if we count on the NTRK classical fusions, only five of 819 patients are with 0.61% incidence rate in CRCs. Though next-generation sequencing (NGS) has been approved as the method for concomitant diagnosis, considering the low prevalence of NTRK fusion and the high cost of testing, it is not the most suitable screening method. Many studies have reported that pan-Trk immunohistochemistry (IHC) is a perfect method for screening NTRK gene rearrangements with high sensitivity and specificity. Previous studies using the antibody of clone EPR 17341 to detect secretory carcinoma and congenital fibrosarcoma harboring NTRK fusions showed a total of 23 cases with NTRK rearrangements detected on MSK-IMPACT were all identified by IHC, suggesting pan-TRK IHC showed the 100% accuracy (24). However, some investigators suggested it was useless to use pan-TRK IHC for the identification of NTRK fusions. They also used antibody of clone EPR 17341 to detect a total of 494 mesenchymal tumors; in 16 IHC staining cases, only four cases showed strong diffuse nuclear and/or cytoplasmatic staining, and one case showing diffuse, but weak cytoplasmic staining was proved harboring NTRK fusion by NGS; the other eleven cases with focal weak and moderate cytoplasmic/membranous or focal moderate to strong nuclear staining did not harbor NTRK fusions (25). As to colorectal cancers (CRCs), nine of 4,569 IHC positive cases demonstrated gene rearrangements by NGS suggesting close to 100% specificity for IHC (26). However, in our study only six pan-TRK IHC positive cases were proved with NTRK fusions by RNA-based NGS; the other four cases were proved non-fusions. When restricting to classical NTRK fusions, four of six cases with pan-TRK IHC positive were proved with classical fusions by RNA-based NGS. Different interpretation scores, antibodies, automation platforms, and dilutions of antibodies are all needed to be evaluated individually in each laboratory (24, 27–29), so IHC is not a reliable method for NTRK fusion screening.

Although fluorescence *in situ* hybridization (FISH) of NTRKs testing is not FDA approved for concomitant diagnosis to our best knowledge, it is the most visual aid for clinical use and usually regarded as the most reliable method for testing genetic translocation (30). Break-apart probes for the three NTRK genes have been used to identify fusions and are commercially available from multiple sources (17, 31–33). In our study, FISH did have high accuracy compared with NGS. In a total 13 FISH positive cases, 12 were proved with NTRK fusions. However, FISH cannot tell the classical, sub-classical, and non-classical fusions of NTRKs. In the total of 13 FISH positive cases, only five cases formed the classical fusion chimeras; the remaining eight cases with either totally or partially missing tyrosine kinase (TK) domain of NTRKs were regarded useless or uncertain in theory when patients were under NTRK fusion-based target treatment. For FISH design, break-apart probes often mark the two sides of NTRKs, the green fluorescence-labeled 3′ and the other red fluorescence-labeled 5′ terminal. Under normal circumstances, the red and green fluorescence are merged by the naked eyes; when genetic translocation occurs the two signals break apart and show separate green and red signals. Theoretically, so long as probes labeling the TK domain have remained, effective fusions are formed. However, even in classical break-apart signal cases, only four of six cases were proved with classical fusions by NGS. Not to mention single signal modes, only two of seven cases harbor classical fusions. Since fusions can involve any partners through either balanced or unbalanced translocation or large deletions, non-effective fusions would not be excluded in FISH testing. To this end, FISH is neither a reliable method for decisive NTRK fusion screening. Different probe designs, different probe lengths, complicated genetic modes, vague cutoff values are all factors that influence the interpretation of FISH results.

Recently many studies reported that the majority of NTRK fusion-positive cases were dMMR (MLH1/PMS2 deficient) which was in accord with our results. In our study, all of the five classical NTRK fusion cases were harboring MLH1/PMS2 deficiency, whereas only one of six sub-classical fusions were dMMR. So when the preliminary screening of FISH showed positive, DNA mismatch repair (MMR) IHC testing was necessary to further tell the true fusions. Whereas, different from other studies that NTRK gene rearrangements are highly enriched in RAS wild-type colorectal carcinomas (26, 34–36), there were only two classical fusion cases harboring KRAS (n = 1) and NRAS (n = 1) mutations; but no BRAF mutation cases were in our study. It is known that approximately 30% of MLH1-hypermethylated BRAF wild-type CRCs harbor KRAS mutations (37). Until now, the relationship between the RAS-MAPK signaling pathway and NTRK rearrangements is unknown.

In western populations, NTRK rearrangements CRCs have a predisposition for right-sided involvement, female predominance, frequent solid growth pattern, mucinous differentiation, and high tumor-infiltrating lymphocytes. Different from these findings, there was no significant difference in any clinicopathological characteristics between NTRK fusion-positive and -negative cases based on the Chinese CRC population. For clinicopathological characteristics, besides MMR (mismatch repair) status (p = 0.001), there is no difference between the NTRK fusion-positive and -negative cases. Nevertheless, classical fusion cases prefer low differentiation (p = 0.001) and different patterns of growth (p < 0.001).

The most common NTRK rearrangements found in the present study involved NTRK1 (n = 7) with four classical fusions, partnered with TPM3 (n = 2), TPR (n = 1), and 5′-telomere (n = 1). Two cases involved NTRK2 with one APBB1IP-NTRK2 classical fusion, one NTRK2-FAM110B sub-classical fusion. All four cases involved NTRK3 rearrangements proved with sub-classical fusion, partnered with ALLC, CCDC73, PBX1, and HOXC13. Besides TPM3 and TPR, other partners reported in our study have not previously been described in any malignancy including CRCs. To date, reported NTRK fusion partners in CRC include LMNA, TPM3, EML4, SCYL3, TPR, and ETV6 (14, 24, 38). Fusions involving all three NTRK genes have shown good response to larotrectinib in a recent basket trial (39). So we concluded that patients carrying any of the classical NTRK fusions should have a good response to TRK-based target treatment, and precisely screenng the classical NTRK rearrangements is crucial to clinical treatment. As only NGS could tell the precise information of break-apart point and fusion modes, it is important for patients to undergo NGS to fully predict the patients’ outcome to larotrectinib treatment. More importantly, RNA-based sequencing is more sensitive than DNA-based sequencing, and only RNA-level fusion provides direct evidence whether these fusions are functionally transcribed and translated. In this study, only three cases were detected with NTRK rearrangements by DNA-based NGS *versus* 13 cases detected by RNA-based NGS. Besides, six cases marked as sub-classical fusions in our study have not been reported in other research, and the therapeutic effect of NTRK-fusion-based target treatment is unknown.

In conclusion, pathogenic NTRK fusions occur in only a small minority of CRCs—estimated at 1.58% in our study with previously reported incidences of 0.16–0.31% (6, 11). Because of their rarity, NTRK fusions can be difficult and expensive to identify in the routine clinical setting. In our study, given the low sensitivity and specificity, Trk IHC is not a reliable method for screening the presence of NTRK rearrangements in CRC. Whereas pan-TRK IHC is pretty much cheaper and more feasible than other methods; especially pan-TRK IHC positive cases have a good chance to be effective fusions, so we still recommend pan-TRK IHC as the primary screening tool. As NTRK fusions (especially classical fusions) are mostly narrowed to MLH1/PMS2 deficiency cases and universal MMR/MSI screening is established as part of routine clinical care in most laboratories, MMR status could also be the first step to screen potential NTRK fusion-positive patients. If MLH1/PMS2 deficiency, we propose addition of FISH in whom TRK-based targeted therapy is being considered. Whereas, when patients harbor sub-classical NTRK fusions with negative pan-TRK IHC and FISH or with pMMR, NGS is the only method to precisely predict the effectiveness of molecular target treatment. Anyway, only RNA-based NGS is the finally clinically validated methodology that determines patients who would benefit from this novel targeted therapy. There also have several limitations in our study. First, due to the single-center experience, the sample size was relatively small. Second, for clinical diagnosis, fusions that only maintain the tyrosine kinase are considered as FISH positive; however, in our study we set all break-apart, single red and signal green signals as FISH positive. And we found that cases with single signals missing the kinase domain of NTRK in theory were often proved as sub-classical fusions by NGS. Third, the therapeutic effect of NTRK fusion-based target to patients harboring sub-classical NTRK fusions is worth further exploring.

## Data Availability Statement

The data presented in the study has been deposited into a publicly accessible repository: http://figshare.com/articles/dataset/_/14446332.

## Ethics Statement

This study was approved by the Ethics Committee of Nanjing Drum Tower Hospital, and all procedures performed in studies involving human participants were in accordance with the ethical standards of the institutional and/or national research committee and with the 1964 Helsinki declaration and its later amendments or comparable ethical standards. Samples were obtained after the patients had provided informed consents.

## Author Contributions

Clinical and pathological analysis: XP, YF, XF, JY, and FG. FISH: XP and ZL. IHC: YF and HW. Next-generation sequencing: XP. Sanger sequencing: XP and BZ. Statistical analysis: XP and ZL. Article writing: XP. Study design: XP, YF, and XF. All authors contributed to the article and approved the submitted version.

## Funding 

This work was supported by grants from the Chinese National Science Foundation (81802394 to XP) and Fundamental Research Funds for the Central Universities (021414380408 to XP).

## Conflict of Interest

The authors declare that the research was conducted in the absence of any commercial or financial relationships that could be construed as a potential conflict of interest.
